# Correlation between muscle architecture and anaerobic power in athletes involved in different sports

**DOI:** 10.1038/s41598-021-92831-7

**Published:** 2021-06-25

**Authors:** Kyu-Lim Lee, Tae-Woong Oh, Young-Chun Gil, Hee-Jin Kim

**Affiliations:** 1 Department of Oral Biology, Division in Anatomy and Developmental Biology, Human Identification Research Institute, BK21 PLUS Project, Yonsei University College of Dentistry, 50-1 Yonsei-ro, Seodaemun-gu, Seoul, 03722 South Korea; 2 Department of Sports Leisure, Yongin University, 134 Yongindaehak-ro, Cheoin-gu, Yongin-si, Gyeonggi-do, 17092 Republic of Korea; 3 Department of Anatomy, Chungbuk National University School of Medicine, 1 Chungdae-ro, Seowon-gu, Cheongju, Chungcheongbuk-do South Korea; 4 Department of Materials Science and Engineering, College of Engineering, Yonsei University, 50 Yonsei-ro, Seodaemun-gu, Seoul, 03722 South Korea

**Keywords:** Anatomy, Health care, Health occupations

## Abstract

Athletes cultivate highly developed muscles based on their sport category, creating a body shape that matches the characteristics of that sports category. We tested the significance of the correlation between muscle development characteristics and anaerobic power in athletes to build a database for each category. Fifty-eight college athletes participated in this study. To assess muscle characteristics, muscle thickness (MT) and fascicle angle (FA) were measured by ultrasonography (US) in lower limb. Furthermore, anaerobic power was measured with the Wingate test. Analysis of the correlation between muscle structure and anaerobic power revealed significant differences between the sports categories, except for the MT of the medial head of gastrocnemius (Gm), lateral head of gastrocnemius, and FA of Gm. A significant difference was observed for all parameters, except for the arrival time to peak power in the anaerobic power items; in particular, a high degree of correlation in mean power/kg and peak power/kg was observed. A similar tendency was observed in the correlation between muscle structure and anaerobic power in most sports categories, but certain muscle characteristic factors were prominent in each sport. Based on these, it is possible to contribute to predicting and promoting athletic performance.

## Introduction

Athletes grow highly developed muscles that work according to the chosen sports category, building a body shape that matches the characteristics of that sport. Compared to non-athletes, shape change is inevitable in athletes who continuously perform high-intensity training. In particular, the lower limbs are the largest muscle group in our body, and because they act on the movements of the hip and knee joints, larger changes can be predicted.

In previous studies, it is known that the performance potential of muscles is improved according to the characteristics of the muscles, and it has been reported that the trained thigh muscle has a higher potential^1^. It has been reported that muscle strength is highly correlated with muscle volume and composition. Previous studies have reported a significant correlation between muscle thickness (MT) and anatomical cross-sectional area^2–5^, and several additional studies have reported that muscle cross-sectional area and muscle volume provide a valuable basis for predicting muscle strength and the exerting force^1,5–8^. Recent studies have shown that the relationship between muscle volume and MT is strongly correlated with muscle force^1,8, 10–13^.Another factor of muscle architecture except MT, the fascicle angle (FA), is correlated with the force exerted by the muscles. The larger the FA, the higher the capacity to pack the contractile material into a certain volume, thus producing strength^14–16^. Muscles are classified according to their shape, and it is known that the pennate muscle can have more muscle fibers per unit area than other kinds of muscle, thereby having the ability to generate large force by providing a larger physiological cross-section^13,17,18^. These muscle morphology directly affect the force production and can manifest differently, depending on the sports category^19^. According to Kanehisa et al., who studied the FA and the MT of swimmers and footballers, the MT of VL and Gm was similar in both groups, but the FA was larger in soccer players. Moreover, it was reported that MT and FA were also positively correlated in both the sports categories^10^ Thus, it can be determined that the muscle structure and morphology can be explained in connection with its muscle strength^19^.

Most of the mentioned studies simply observed the morphology of the muscle, had limited sports categories, or targeted only a few muscles. In addition, studies on the correlation between the morphological characteristics of the muscles and motor performance in different sports categories have been insufficient. Furthermore, these previous studies have used medical imaging methods, such as computed tomography (CT) and magnetic resonance imaging (MRI)^9,20^. Although CT or MRI can provide valuable information, their cost is high, and the results are slow to process. In contrast, ultrasonography (US) is faster, easier, and more economical; it also has high stability, thus reducing the burden on patients. In this respect, US has been suggested as the most appropriate method for describing muscle architecture in vivo^19,21^^.^ However, it has not been widely applied in many cases.

The purpose of this study was to identify the characteristics of muscle development and to determine whether such characteristics are significantly correlated with anaerobic performance. Furthermore, the aim was to compare and propose patterns observed in accordance with each sport category and, thus, build a physical-development database of athletes based on the morphological characteristics of the muscles and anaerobic power. The results of this study can be applied to coaches developing training programs that include resistance training based on the characteristics of each sport category and these programs can promote prediction and improvement of performance.

## Materials and methods

Fifty-eight male volunteers [mean age, 20.1 ± 1.4; height, 175.6 ± 6.7 cm; weight, 77.3 ± 17.2 kg; body mass index (BMI), 22.5 ± 7.1; mean ± standard deviation (SD)] were recruited in this study. Among them were 10 boxers, 8 were Judo athletes, 10 were Taekwondo athletes, 10 were soccer players, 10 were wrestlers, and 10 were traditional Korean wrestlers. All of them were college athletes who had more than five years of training experience and constantly carried out high-intensity training; they also participated in several national competitions. None of the participants had any relevant medical history or injuries in the anterior thigh and lower leg region and had no factors that could affect or interfere with any of the procedures or examinations.

Before conducting this study, all participants were given a detailed description of the purpose, methods, and risks of the study and were informed that if they so desired, they could withdraw from the experiment at any time. Following this, they signed an informed consent document. The study was approved by the Ethics Committee of Yongin University (IRB No.: 2-1040966-AB-N-01-20-1908-HMR-145-4), and the study was performed in accordance with the Declaration of Helsinki.

### Ultrasonography examination

All US examinations to measure the MT and FA were performed using a real-time two-dimensional scanner (Minisono, ALPINION Medical Systems, Seoul, Korea) with a B-mode high-frequency linear array transducer (8.017 MHz; L3-12, ALPINION Medical Systems).

**Figure 1:**
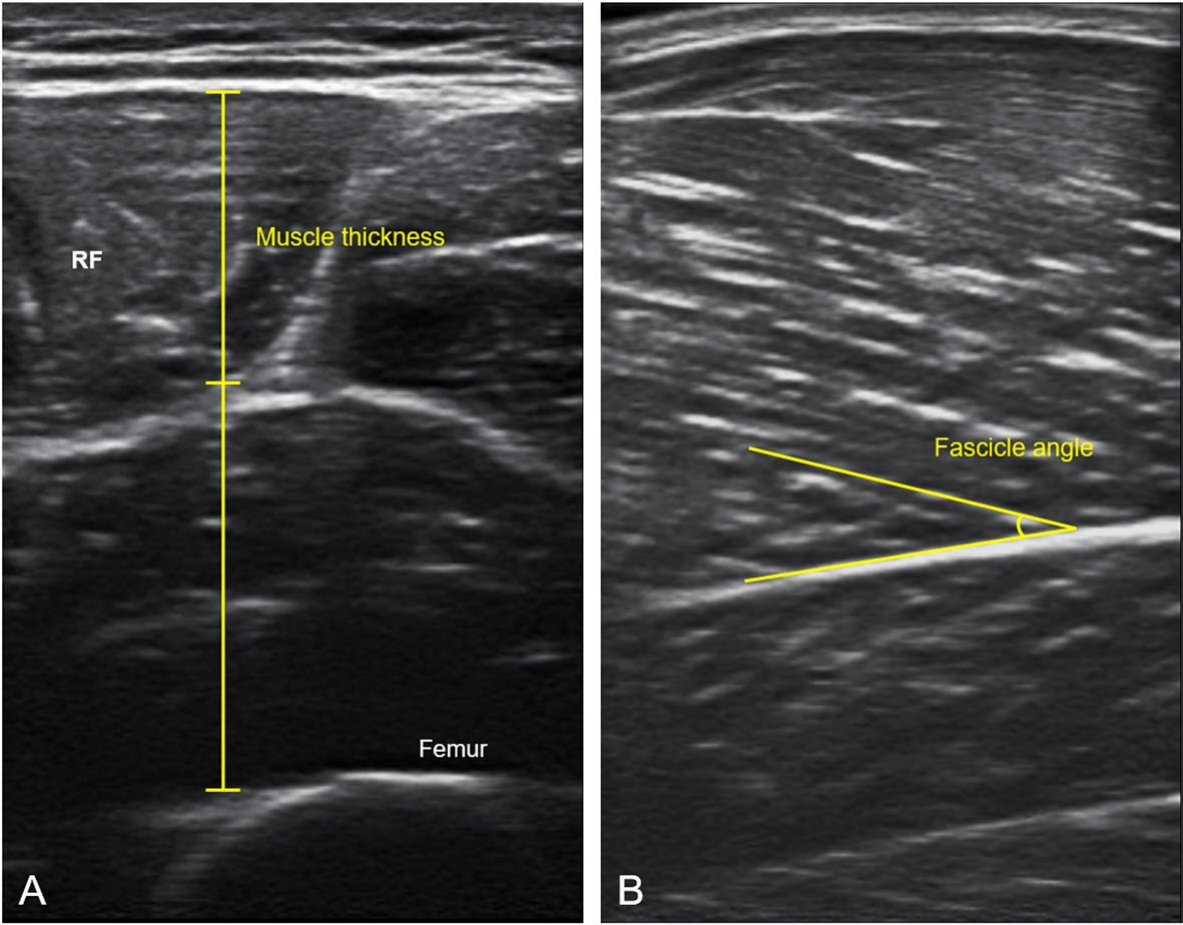
Representation of muscle architecture measurement of rectus femoris (RF) at the sonographic image **(A)** muscle thickness; **(B)** fascicle angle.

The participants were placed in a standing position throughout the procedure. US examination was performed in the anterior and lower leg regions. The MT of the rectus femoris (RF) on the superior 30% and 50% levels between the anterior superior iliac spine and the superior border of the patella, vastus medialis (VM), vastus lateralis (VL) at the midpoint between the superior 50% level of RF and the superior border of the patella in the anterior thigh region, and tibialis anterior (TA) on the superior 30% between the fibular head and lateral malleolus, medial head of gastrocnemius (Gm), and lateral head of gastrocnemius (Gl) on the most prominent point in the lower leg region were measured. The most prominent points were identified using a tape measure. US images in the transverse and longitudinal planes were obtained at each point, and the FA values of the RF, VL, Gm, and Gl were measured based on the bony landmarks. Image analysis software (ImageJ; National Institutes of Health, Bethesda, MD, USA) was used to determine the MT and FA in the acquired US images (Fig. 1).

### Wingate test

The device used in the present study was an electromagnetic ergometer (Computer-aided electrically braked cycle ergometer; Lode B. V. Excalibur Sports, Netherlands) with a given torque of 0.075 kg per weight^22,23^. All procedures were measured and recorded using Lode Wingate version 1.0.7 software (Lode B. V. Excalibur Sports, Netherlands). After Wingate test, 10 s, 20 s, 30 s power, peak power (w), arrival time (sec), peak power (w/kg), mean power (w), and mean power (w/kg) were measured, respectively.

In this study, the ergometer was set to 60 s for the warm-up stage (slow pedaling), 30 s for the sprint stage (quick and powerful pedaling), and 60 s for the cool-down stage (slow pedaling). Participants then engaged with the ergometer, and measurements were taken during each consecutive stage of the procedure. The purpose of the cool-down stage was to promote the safety of the participants.

To inspire competition, more than two sports-category athletes were examined at a time, and continuous moral support was promoted to encourage participants to demonstrate their maximum power.

### Statistical analyses

All statistical analyses were conducted using standard statistical software (SPSS version 23.0, IBM Corp., Armonk, NY, USA). The probability cutoff for statistical significance was set at P < 0.05. One-way ANOVA for f, the MT and FA data for each muscle were compared to the anaerobic force for each sport category to determine their significance. Subsequently, Pearson's correlation to understand the correlation between muscle structure and anaerobic force. The correlation coefficient and significance probability were confirmed, and the significant value was confirmed at the 0.05 level. In addition, verification through stepwise multiple linear regression analysis was performed. Through this, a significant structure for each event was calculated and determined as the core muscle of the corresponding event.

## Results

### Mean values of muscle architecture and anaerobic power of all athletes

In the anterior thigh region, the MT of RF (30%, 50% level) was 29.18 ± 4.22 mm and 29.10 ± 4.40 mm, respectively, with no significant difference between the levels; the FA of RF (50% level) was 12.90° ± 4.45°. The MT of the VM was 32.95 ± 6.11 mm, and the VL thickness and FA were 27.61 ± 5.17 mm and 14.56° ± 4.26°, respectively. In the lower leg region, the MT of the TA was 31.36 ± 4.70 mm. In addition, the MT of gastrocnemius was 21.31 ± 3.51 mm in medial head and 14.30 ± 2.73 mm in lateral head, whilst the FA was 21.34° ± 3.00° and 13.68° ± 3.96°, respectively. In both cases, Gm showed a higher value than Gl (Table 1).

**Table 1:** Mean values of the muscle thickness, fascicle angle and anaerobic power measurements of athletes.

*N* = *58*		Muscle thickness (mm)	Fascicle angle (°)
RF	30%	29.18 ± 4.22	–
	50%	29.10 ± 4.40	12.90 ± 4.45
VM		32.95 ± 6.11	–
VL		27.61 ± 5.17	14.56 ± 4.26
TA		31.36 ± 4.70	-
Gm		21.31 ± 3.51	21.34 ± 3.00
Gl		14.30 ± 2.73	13.68 ± 3.96
**Anaerobic power**			
Maximum anaerobic power	Peak power (w)	966.5 ± 161.9	
	Arrival time (s)	5.0 ± 0.9	
	Peak power (w/kg)	14.3 ± 2.0	
Mean anaerobic power	Mean power (w)	589.3 ± 55.1	
	Mean power (w/kg)	8.7 ± 0.5	

Data are presented in mean ± SD values; Arrival time, arrival time to peak power.

The peak power was 1136.69 ± 256.95 W, and the time to reach it was 4.75±0.73 s. The calculation of maximum power based on body mass was 15.18 ± 2.17 W. The mean power was 666.39 ± 89.57 W, and the average power based on body mass was 8.40 ± 1.20 W (Table 1).

### Athletes' muscle architecture and anaerobic power—comparison according to sport category

Among the five muscles analyzed by one-way ANOVA analysis, there was a statistically significant difference in each sport category, except for the MT and FA of Gm and MT of Gl. According to the results, the MT of RF (30%, 50% level), VM, VL, TA, and FA of RF and Gl all showed significant differences by sports category (p < 0.05) (Table 2).

**Table 2:** Muscle structure and anaerobic power measurements by six sport categories.

		Boxing		Judo		Taekwondo		Soccer		Wrestling		Traditional Korean wrestling	
		MT (mm)	FA (°)	MT (mm)	FA (°)	MT (mm)	FA (°)	MT (mm)	FA (°)	MT (mm)	FA (°)	MT (mm)	FA (°)
RF	30%	26.8 ± 3.8	–	31.4 ± 2.8	–	25.7 ± 3.6	–	30.5 ± 3.5	–	28.7 ± 4.0	–	32.0 ± 2.9	–
	50%	26.9 ± 3.2	12.4 ± 1.1	32.0 ± 1.9	15.2 ± 5.8	24.5 ± 3.2	8.7 ± 2.1	31.5 ± 2.7	15.1 ± 3.3	28.0 ± 5.2	17.8 ± 2.9	31.9 ± 3.0	10.6 ± 2.9
VM		29.6 ± 3.1	–	38.3 ± 2.7	–	28.8 ± 4.4	–	38.5 ± 3.8	–	34.4 ± 7.6	–	29.5 ± 4.2	–
VL		25.1 ± 2.2	13.1 ± 2.3	30.8 ± 0.9	18.0 ± 5.2	21.9 ± 5.1	10.8 ± 2.6	31.8 ± 4.1	16.0 ± 3.0	25.8 ± 5.0	16.4 ± 3.5	30.4 ± 1.7	14.6 ± 4.4
TA		33.5 ± 3.2	–	33.9 ± 4.8	–	24.7 ± 4.2	–	32.6 ± 3.0	–	30.5 ± 2.6	–	33.1 ± 2.5	–
Gm		19.8 ± 2.9	20.6 ± 2.6	22.1 ± 3.6	21.2 ± 1.5	20.7 ± 2.2	20.6 ± 3.5	20.9 ± 2.2	23.0 ± 2.5	19.6 ± 3.3	19.6 ± 3.0	24.0 ± 4.1	22.3 ± 2.9
Gl		11.8 ± 1.1	9.5 ± 2.3	15.4 ± 2.7	14.1 ± 3.9	14.2 ± 3.2	17.3 ± 4.2	14.8 ± 2.7	13.9 ± 3.6	15.2 ± 1.7	12.5 ± 1.9	14.4 ± 2.5	13.6 ± 2.2
Maximum anaerobic power	Peak power (w)	966.5 ± 161.9		1284.6 ± 265.6		1138.0 ± 224.8		1189.8 ± 140.1		1102.3 ± 92.7		1283.4 ± 138.2	
	Arrival time (s)	5.0 ± 0.9		4.5 ± 0.6		4.3 ± 0.4		4.8 ± 0.6		5.2 ± 0.9		4.6 ± 0.7	
	Peak power (w/kg)	14.3 ± 2.0		13.9 ± 2.1		16.4 ± 1.7		16.8 ± 1.0		7.0 ± 0.5		13.5 ± 2.2	
Mean anaerobic power	Mean power (w)	589.3 ± 55.1		716.4 ± 91.9		624.4 ± 81.6		699.9 ± 49.8		615.9 ± 52.1		754.9 ± 63.1	
	Mean power (w/kg)	8.7 ± 0.5		7.8 ± 0.9		9.1 ± 0.6		9.9 ± 0.5		16.4 ± 1.0		7.9 ± 1.1	

Data are presented as mean ± SD values.

*MT* muscle thickness, *FA* fascicle angle, *RF* rectus femoris, *VM* vastus medialis, *VL* vastus lateralis, *TA* tibialis anterior, *Gm* medial head of gastrocnemius, *Gl* lateral head of gastrocnemius.

In the anterior thigh region, the mean-value ranges of MT of the RF (30% and 50% level) in the six sports categories were 25.7–31.4 mm and 24.5–32.0 mm, respectively; and the range of the FA (50% level) was 8.7°–17.8°. The range of VM thickness was 28.8–38.5 mm; and the ranges of VL thickness and FA were 21.9–31.8 mm and 10.8°–18.0°, respectively. In the lower leg region, the range of MT of the TA was 24.7–33.9 mm. For Gm, the MT and FA ranges were 19.6–24.0 mm and 9.6°–17.3°, respectively; and for Gl, the MT and FA ranges were 11.8–15.4 mm and 9.5°–17.3°, respectively (Table 2).

One-way variance analysis among anaerobic analysis values revealed a statistically significant relationship between sports categories in terms of peak power, peak power/kg, average power, and average power/kg (excluding arrival time only) at 10 s, 20 s, and 30 s (p<0.05). In the early stages of the Wingate test, the highest value was observed in traditional Korean wrestlers, but a decreasing trend was observed over time. Judo athletes showed the strongest peak power; however, the soccer players showed the shortest time to reach peak power, as well as the highest peak power against weight value. In the case of mean power, traditional Korean wrestlers were the strongest, but wrestlers demonstrated exceptional strength in terms of mean weight (Table 2).

A significant difference from post-hoc comparison analysis between sport categories was observed in all values except in MT of Gm; Gl; and in FA of Gl; and in arrival time to peak power. The results showed a strong difference between almost all sports categories, especially when analyzing the peak and mean powers that reflected the weight (p < 0.05).

### Correlation between muscle architecture and anaerobic power of athletes according to sport category

The R-value between the mean power and RF thickness (50% level) was 0.592, showing the strongest positive correlation among all muscles. FA of RF values were not significantly correlated with the exertion of anaerobic power. The peak power was also related to the RF thickness. No significant differences were observed in the body weight values. The peak power and mean power also showed a relationship between the Gm thickness and FA. The R-value between the mean power and Gm thickness was 0.498, showing the strongest positive correlation (p < 0.01) (Table 3). Likewise, the peak power (kg) showed a highly significant relationship with TA thickness (Table 3).

**Table 3:** Coefficients for Pearson’s correlations between muscle thickness and fascicle angle of lower leg region muscle and anaerobic power.

Variable		RF 30%	RF 50%		VM	VL		TA	Gm		Gl	
		MT (mm)	MT (mm)	FA (°)	MT (mm)	MT (mm)	FA (°)	MT (mm)	MT (mm)	FA (°)	MT (mm)	FA (°)
10 s (w)	*r*	0.339*	0.334*	0.054	0.316*	0.300*	0.095	−0.090	0.172	0.176	0.253	0.214
20 s (w)	*r*	0.431**	0.498**	0.091	0.178	0.292*	0.239	0.227	0.488**	0.385**	0.016	−0.224
r30s (w)	*r*	0.196	0.349*	0.105	0.377**	0.211	0.080	0.131	0.349*	0.305*	−0.026	−0.306*
Peak power (w)	*r*	0.277*	0.321*	−0.067	0.270	0.162	−0.015	−0.060	0.405**	0.243	0.161	0.121
Arrival time (sec)	*r*	−0.020	−0.127	0.143	0.077	−0.021	0.046	0.137	−0.069	−0.023	0.097	−0.122
Peak power (w/kg)	*r*	−0.106	−0.124	0.041	0.261	−0.153	−0.074	−0.430**	0.055	0.071	0.211	0.087
Mean power (w)	*r*	0.516**	0.592**	0.029	0.335*	0.371**	0.185	0.028	0.498**	0.360**	0.147	−0.015
Mean power (w/kg)	*r*	–0.227	−0.176	−0.214	0.057	−0.040	−0.090	−0.174	0.094	0.243	−0.032	−0.009

*R* R-value; significantly different, *p < 0.05, **p < 0.01, *RF* rectus femoris, *VM* vastus medialis, *VL* vastus lateralis, *TA* tibialis anterior, *Gm* gastrocnemius medial head, *Gl* gastrocnemius lateral head, *MT* muscle thickness, *FA* fascicle angle.

Pearson’s correlation coefficient results indicated that a significant relationship among the RF, VM, and VL was largely shown in the anaerobic power evolution of the initial stage. The peak power and mean power showed a complex correlation between the anterior thigh and lower leg region muscles (p < 0.05). This result showed a similar pattern to the results of the statistical analysis before classification into sports categories. This suggested that in all athletes, there was a correlation between muscle architecture and anaerobic power, regardless of the sports category (Table 3).

The stepwise multiple linear regression analysis results are as follows: Multiple regression analyses revealed that the MT of VM and Gm is a significant factor in predicting peak power, the variance analysis of statistical significance showed a p-value of 0.004, the MT of RF (50%), and Gm was a significant factor in predicting mean power; and the variance analysis of statistical significance showed a p-value of 0.002 (Table 4).

**Table 4:** Screening of explanatory models using stepwise multiple linear regression analysis.

Variable		B	t	P-value
20 s power	TA MT	5.444	2.076	0.045*
	Gm MT	11.947	3.855	0.000**
30 s power	VM MT	6.234	3.387	0.002**
	Gm MT	10.614	3.000	0.005**
	Gl MT	− 11.689	− 2.585	0.014*
Peak power	VM MT	14.660	2.240	0.031*
	Gm MT	35.522	2.966	0.005**
Peak power/kg	VM MT	0.194	4.189	0.000**
	VL MT	−0.129	−2.486	0.018*
	TA MT	−0.141	−2.038	0.049*
Mean power	RF (50%) MT	8.292	3.253	0.002**
	Gm MT	3.263	3.260	0.002**

*B* unstandardized coefficient, *t* t statistic; significantly different, **p < 0.01, *p < 0.05, *MT* muscle thickness, *RF* rectus femoris, *VM* vastus medialis, *VL* vastus lateralis, *TA *tibialis anterior, *Gm* medial head of gastrocnemius, *Gl* lateral head of gastrocnemius.

## Discussion

In athletes, the muscles responsible for the movement of specific joints that are repeatedly exercised become thicker. Such muscles can be used as indicators to predict athletes' performance and to obtain information about their functional ability. Various studies have reported that anatomical cross-sectional area and muscle-volume data can be used to predict strength^2,3,6,7,9,24^. In addition, it has been reported that a combination of MT and morphological limb length is useful for estimating muscle volume^10^. Through these prior studies, it can be inferred that the developmental form or structure of the muscle directly affects exercise power. In previous studies, CT and MRI were used to measure muscle volume and anatomical cross-sectional area, and most studies used EMG measurement using electromyography to measure the muscle strength^9,25^. Most previous studies performed and reported only morphological comparisons using results of simple observation of muscles^2,26^. In addition, there are few reports on the association between muscle and specific sports ability. Thus, it was not possible to determine whether this result could explain the general relationship for various sports. A previous study compared the differences in muscle structure between swimmers and soccer players. The VL thickness was thicker in swimmers than in soccer players, and there was no significant difference in Gm thickness between the two samples. VL thickness and fascicle length were significantly correlated in both these sports categories^10^. Based on this, the authors confirmed the need for further investigation into the characteristics of each sports category. Therefore, in this study, we attempted to determine the relationship between muscle architecture and anaerobic power in six categories of athletes.

According to the results, there was a statistically significant difference between the muscle structure and various measurement parameters. Moreover, a similar tendency was observed in most sports categories. The muscle development architecture of the anterior thigh region showed a significant correlation with most of the anaerobic power metrics, and the MT contribution to the exertion of anaerobic power was especially high.

The interpretation of each result is as follows:

The mean thickness of the RF was approximately 29 mm, and no significant difference was observed at 30% and 50% levels of RF. The average VM and VL values were 33 and 28 mm, respectively. The TA had a mean thickness of 31 mm, and the gastrocnemius was 21 mm and 14 mm in the medial and lateral head, respectively. In addition, MT and FA were higher in Gm than in Gl.

Athletes’ muscle architecture and anaerobic power comparison based on sport category showed statistically significant differences between each sports category in all muscle items except the gastrocnemius. Upon comparing the anaerobic power items, a statistically significant difference was observed among all values except for the arrival time to peak power. In the early stages of the Wingate test, traditional Korean wrestlers showed the highest values, but a decreasing trend was observed over time. In addition, Judo athletes showed the highest peak power; however, soccer players showed the shortest time to reach peak power and the strongest peak power per weight. Wrestlers showed the highest mean power value but were mean power per weight, showing a slight drop in the value.

In recent studies, VL has been reported to be the muscle, from among the thigh muscles, that contributes most to the exertion of propulsion^13,27^. However, according to the results of the present study, it is apparent that the main muscle responsible for reaching maximum power can differ according to the category of the athlete.

This study demonstrated a clear correlation between muscle characteristics and anaerobic power in athletes. This finding was corroborated by a previous report that found that the physiological cross-sectional area and the maximum power generation capacity increased with an increase in the MT and FA^2–5,13^. According to the results of the present study, the factors that influence the exertion of maximum power in athletes are the MT of the RF, VL, and gastrocnemius, and the FA of RF and Gm. The results analyzed according to the six sports categories showed similar tendencies to those obtained without classifying them into categories.

Based on these results, coaches can develop training programs that include resistance training based on the characteristics of each sports category. Programs designed to intensively develop the maximum power exerting muscles for sports that require explosive power, and to intensively develop the mean power exerting muscles for sports that require endurance, can be expected to improve athletes' performance. In addition, such programs can facilitate prediction and improvement of performance.

The correlation between muscle architecture and the anaerobic power of athletes according to sports category was analyzed in this study. The results analyzed according to the six sports categories showed similar tendencies to those obtained without classifying them into categories. The main findings of this study are as follows.

**Figure 2:**
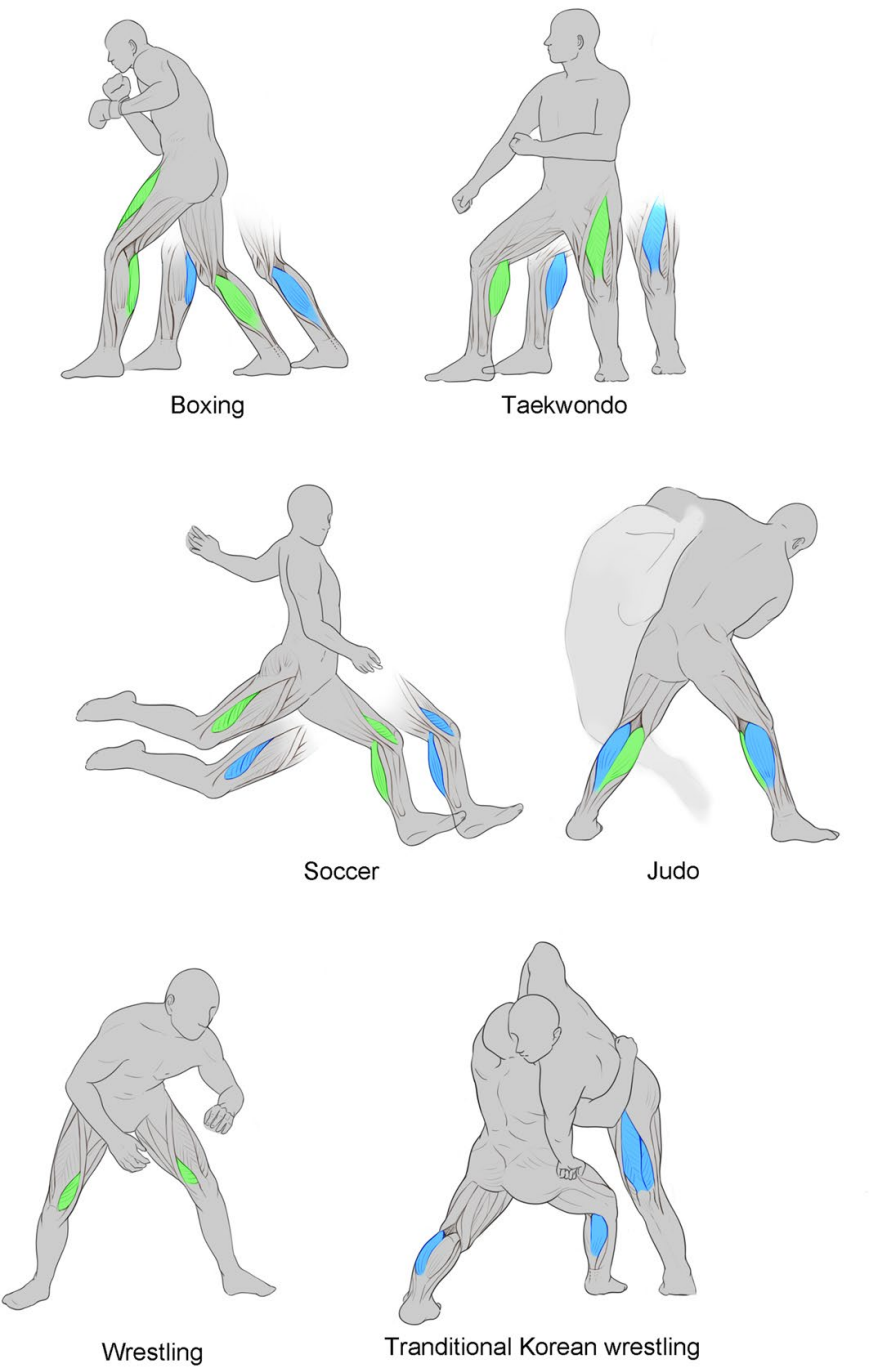
Main muscles involved in reaching for maximum power and mean power in Athletes from different sports categories. Peak power related muscles are indicated in blue color; Mean power related muscles are indicated in green color.

- 1. In the case of boxers, the factor that contributes to the maximum power is the thickness of Gm. The thicknesses of the Gm and RF work together to exert the mean power.
- 2. In the case of Judo athletes, the MT of Gl contributes to the maximum power and the MT of Gm contributes to the mean power.
- 3. In the case of Taekwondo athletes, the MT of RF and Gm acts together to exert maximum power and mean power.
- 4. In the case of soccer players, the MT of VL and the FA of Gm are common factors contributing to both maximum and mean power.
- 5. In the case of wrestlers, a statistically significant relationship was found between the MT of VM and mean power exertion. No statistical significance was observed between maximum power and muscle structure.
- 6. In the case of traditional Korean wrestlers, the FA of the RF and MT of VL and Gl were found to be factors that contribute to reaching maximum power exertion. No statistical significance was observed between mean power and muscle structure.
- Therefore, on this basis, it can be concluded that the muscles that contribute to reaching the maximum power and mean power in each sport can be predicted accordingly. (Fig. 2).
